# Autoimmune Pancreatitis Mimicking Obstructive Pancreatic Neuroendocrine Tumor

**DOI:** 10.7759/cureus.64248

**Published:** 2024-07-10

**Authors:** Rakahn Haddadin, Amit Grewal, Srusty Patel, Zachary I Merhavy, Homayon Iraninezhad

**Affiliations:** 1 Medicine, MountainView Hospital, Las Vegas, USA; 2 Internal Medicine, MountainView Hospital, Las Vegas, USA; 3 Neurology, St. George's University, Miami, USA; 4 Cardiology, Dell Seton Medical Center, Austin, USA; 5 Gastroenterology, MountainView Hospital, Las Vegas, USA

**Keywords:** endoscopy ercp, medical intensive care unit (micu), clinica gastroenterology, pancreatic neuroendocrine tumors, autoimmune pancreatitis (aip)

## Abstract

Autoimmune pancreatitis (AIP), otherwise known as non-alcoholic destructive pancreatitis or sclerosing pancreatitis, is a rare form of chronic pancreatitis that is clinically significant due to its potential to mimic pancreatic cancer. In our case, we present a 64-year-old male with a past medical history of type 2 diabetes and epigastric abdominal pain for one year who presented with worsening epigastric abdominal pain, 12-pound weight loss, and vomiting and was found to have a neuroendocrine tumor on a preliminary pathology report, while official pathology later came back stating AIP. Distinguishing between autoimmune pancreatitis (AIP) and pancreatic cancer is vital, given the stark contrast in their treatment and prognosis. In our case, preliminary pathology suggested a neuroendocrine tumor, prompting consultation with oncology. Utilizing invasive testing like EUS-FNA, we obtained an official diagnosis and prevented the patient from undergoing unnecessary treatments and interventions. Our case shows the importance of further testing when a patient presents with a fast-growing obstructive pancreatic mass. While searching the literature, there are no previously documented cases of an AIP mass as large as our patients and as fast-growing.

## Introduction

Autoimmune pancreatitis (AIP), otherwise known as non-alcoholic destructive pancreatitis or sclerosing pancreatitis, is a rare form of chronic pancreatitis that is clinically significant due to its potential to mimic pancreatic cancer [1]. AIP has been distinguished between AIP-1, which is considered a disease mediated by an IgG4-related immune system assault on the pancreatic tissue, and AIP-2, which is a pancreas-specific disease unrelated to IgG4 [1,2]. Initiating mechanisms for AIP have been noted to include bacterial infection and molecular mimicry in the setting of genetic risk factors and autoimmunity [3]. This autoimmune reaction triggers inflammation and fibrosis within the pancreas, often resulting in pancreatic insufficiency and dysfunction, as the IgG4 antibodies are thought to behave as tissue-destructive immunoglobulins [3].

AIP is often characterized by obstructive jaundice with or without pancreatic masses, lymphoplasmacytic infiltrate and fibrosis, and a marked response to steroids [1]. Therefore, distinguishing AIP from pancreatic cancer poses a complicated challenge, as aspects of both patient presentation and imaging are not always distinct enough to confirm a diagnosis. As other similar cases are seldom reported, understanding how to treat and identify this condition is only slowly becoming more defined. The nosology of AIP is complex and not yet perfected. Thus, further discussion on cases of the condition will lead to a better understanding of diagnosis and treatment in the future [1,3]. This report presents one such rare case of AIP mimicking a fast-growing obstructive pancreatic cancer. It explores the diagnostic work-up that led to the successful diagnosis of the condition.

## Case presentation

A 64-year-old male with a past medical history of type 2 diabetes and epigastric abdominal pain for one year presented with worsening epigastric abdominal pain, 12-pound weight loss, and vomiting. The physical exam was negative for any jaundice or scleral icterus. Laboratory results are shown in Table 1.

**Table 1:** Patient's lab results during admission

Variable	Value (Reference range)
Alanine transaminase (ALT)	497 g/dl (12-78)
Aspartate aminotransferase (AST)	647 g/dl (15-37)
Alkaline phosphatase (ALK)	157 g/dl (45-117)
Total bilirubin	1.1 g/dl (0.1-1.0)
Lipase	249 g/dl (13-75)
Carcinoembryonic antigen (CEA)	2.7 ng/mL (0.0 - 4.7)
Cancer antigen (CA) 19-9	35 Units/mL (0-35)

**Figure 1:**
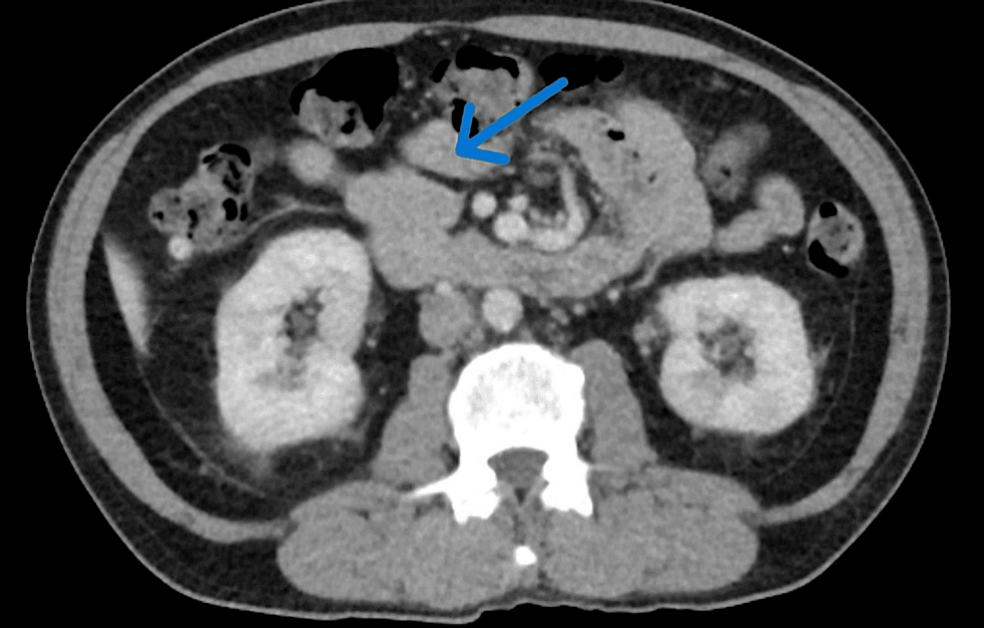
MRI showing the enlarged pancreatic mass that was in question

The patient had a recent outpatient computed tomography (CT) and magnetic resonance imaging (MRI) of the abdomen two months before presenting to the emergency department, which was negative for any pancreatic mass. However, inpatient imaging showed an MRI with an 8.4 x 2.5 x 2.3 cm pancreatic mass that was concerning for pancreatic cancer versus autoimmune pancreatitis (Figure 1). High-grade stenosis of the superior mesenteric vein (SMV) and innumerable bilateral renal lesions were also found. Given renal lesions, concern for IgG was also noted. Gastroenterology, rheumatology, and oncology were consulted.

An endoscopic ultrasound (EUS)-guided fine needle aspiration (FNA) was performed, and preliminary results were positive for a possible neuroendocrine tumor. The sample was sent for an official pathology report. Treatment was held until the final pathology was reported. The patient’s liver function tests were downtrending to the normal range, and the patient was symptom-free before discharge. Serum IgG4 was 466 mg/dL (reference range 2-96), and the official pathology report showed hypocellular and a few benign ductal epithelial cells along with fragments of fibrous tissue. No tumor cells were identified. The diagnosis of autoimmune pancreatitis was confirmed.

Due to the patient's condition being stable, symptoms being resolved, and proper outpatient follow-up arranged, the patient was discharged home safely.

## Discussion

Distinguishing between autoimmune pancreatitis (AIP) and pancreatic cancer is vital, given the stark contrast in their treatment and prognosis. In our case, preliminary pathology suggested a neuroendocrine tumor, prompting consultation with oncology. Utilizing invasive testing like EUS-FNA, we obtained an official diagnosis and prevented the patient from undergoing unnecessary treatments and interventions. 

Individuals with autoimmune pancreatitis typically present with obstructive jaundice with mild abdominal pain that results from sclerosing cholangitis [4]. The predominant demographic affected by AIP is elderly men presenting with severe epigastric pain with elevated IgG4 levels in AIP type 1, as seen in this patient. In histological reports of the pancreas with AIP, the presence of T-lymphocytes, IgG4-positive plasma cells, storiform fibrosis, and occasional obliterative phlebitis can be noted [5]. The sclerosis in the surrounding tissue disrupts the normal function of pancreatic enzymatic secretion, leading to disease processes of the kidneys, swelling of the salivary glands, and biliary tract strictures.

In order to diagnose AIP, there are five cardinal features that must be present: pancreatic histology, imaging of pancreatic parenchyma and ducts, serology with possible elevated IgG4, multiple organ involvement, and an optional response to steroid therapy [6]. The management and treatment of AIP are strongly guided by the response to steroid therapy. Initially, if the patient is jaundiced, transhepatic or endoscopic drainage of stagnant bile takes place. Oral prednisone is administered for up to four weeks and tapered over the next two weeks [7]. In cases where steroids are contraindicated, rituximab is typically administered as monotherapy. If the response is refractory to the treatment, it is important to check the patient for a possible pancreatic tumor. In our case, our patient responded appropriately to steroid treatment.

Pancreatic endocrine tumors (PNETs) are one of the most common groups of neuroendocrine tumors arising from pancreatic tissue that can be classified as functioning or non-functioning, depending on their ability to excrete hormones. Patients with PNETs have a diverse range of clinical features depending on the location and the origin of the tumor. The patients present with abdominal discomfort or pain, jaundice if it is an obstructive mass, weight loss, nausea, vomiting, changes in bowel habits with hypersecretion syndrome, hypoglycemia, flushing of the skin, and a possible palpable mass. 

Nonfunctioning PNETs often have a more convoluted diagnosis that relies on symptoms stemming from tumor mass effect, and the quality and quantity of hormone production by the tumor do not produce a distinct syndrome as seen in a functioning PNET [8]. Obtaining a EUS-FNA has a sensitivity of 79-100%, and additional biochemical marker scans are required to confirm the diagnosis. This includes plasma chromogranin A levels, pancreatic polypeptide (PP), pancreastatin, neuro-specific enolase levels, and somatostatin receptor scintigraphy [9]. On CT with contrast, PNETs have more hypervascularity with heterogeneous cystic changes, calcifications, and irregular necrosis. The treatment strategy for PNETs follows a more aggressive approach, including surgical resection, locoregional radio ablation to metastatic sites, systemic therapy for residual disease, and complication control [10].

It is crucial to optimize the diagnostic workup of such diseases to prevent misdiagnosis and subject the patient to aggressive treatment strategies. Since PNETs and AIP cause obstructive jaundice, the nature of the obstructive jaundice should be further evaluated. In AIP, the obstructive jaundice is variable and does not necessarily progress or regress. In PNETs, the obstructive jaundice progresses as the tumor grows in size. Multiorgan manifestations should also suggest AIP, as symptoms such as swelling of the salivary glands, sclerosing cholangitis, hydronephrosis, and other presentations are not native to PNETs unless there is a potential presence of metastasis.

## Conclusions

Our case shows the importance of further testing when a patient presents with a fast-growing obstructive pancreatic mass. While searching the literature, there are no previously documented cases of an AIP mass as large as our patients and as fast growing.
